# Tocolytic Therapy Inhibiting Preterm Birth in High-Risk Populations: A Systematic Review and Meta-Analysis

**DOI:** 10.3390/children10030443

**Published:** 2023-02-24

**Authors:** Noyuri Yamaji, Hitomi Suzuki, Kana Saito, Toshiyuki Swa, Fumihiko Namba, Joshua P. Vogel, Jenny A. Ramson, Jenny Cao, Lavin Tina, Erika Ota

**Affiliations:** 1 Global Health Nursing, Graduate School of Nursing, St. Luke’s International University, Tokyo 104-0044, Japan; 2 Saitama Medical Center, Saitama Medical University, Saitama 350-0495, Japan; 3 School of Allied Health Sciences, Osaka University, Osaka 565-0871, Japan; 4 Maternal, Child and Adolescent Health Program, Burnet Institute, Melbourne 3004, Australia; 5 UNDP/UNFPA/UNICEF/WHO/World Bank Special Programme of Research, Development and Research Training in Human Reproduction (HRP), Department of Sexual and Reproductive Health and Research, World Health Organization, 1211 Geneva, Switzerland; 6 Tokyo Foundation for Policy Research, Tokyo 106-0032, Japan

**Keywords:** tocolytic therapy, preterm birth, extremely preterm birth, multiple gestations, growth-restricted foetuses, systematic review

## Abstract

This systematic review aimed to identify the benefits and possible harms of tocolytic therapy for preterm labour management in the context of pregnant women with extremely preterm birth, multiple gestations, or growth-restricted foetuses. A comprehensive search using MEDLINE, Embase, the Cochrane Central Register of Controlled Trials, CINAHL, and the WHO Global Index Medicus databases was conducted from 10 to 15 July 2021. We included randomized controlled trials and non-randomized studies that assessed the effects of tocolysis compared with placebo or no treatment. We found 744 reports and, finally, nine studies (three randomized controlled trials and six cohort studies) pertaining to women with <28 weeks of gestation were included. No eligible studies were identified among women with a multiple pregnancy or a growth-restricted foetus. A meta-analysis of the trial data showed that there were no clear differences in perinatal death nor for a delay in birth. Non-randomized evidence showed that tocolysis delayed birth by 7 days, though there was no clear difference for preterm birth. In summary, it remains unclear whether tocolytic therapy for inhibiting preterm labour is beneficial for these subgroups of women and their newborns. Further well-designed randomized controlled trials and observational studies are needed to address the lack of evidence regarding tocolytic agents in these populations.

## 1. Introduction

The World Health Organization (WHO) defines preterm birth as a live birth that occurs before 37 weeks of gestation [1]. In 2014, the global burden of preterm birth was estimated at 14.84 million babies [2]. It is estimated that over 60% of preterm births occur in Africa and South Asia; preterm birth rates appear to be rising in many countries. Preterm birth complications are the primary cause of death in children under 5 years of age, though the majority of these deaths could be prevented with universal access to good-quality maternal and preterm newborn care [3,4]. Preterm birth is classified into three categories based on gestational age: extremely preterm (<28 weeks), very preterm (28 to 32 weeks), and moderate to late preterm (32 to 37 weeks) [1]. Among these groups, extremely preterm neonates have the highest rates of mortality and morbidity, with more than 90% of these infants succumbing in low-income countries with limited resources [5]. Despite the fact that the majority of preterm births do not exhibit a definitive risk factor [6], a portion can be attributed to medical conditions and pregnancy complications [4]. Multiple pregnancies and growth-restricted foetuses are considered high-risk factors for preterm birth and are associated with higher rates of perinatal morbidity and mortality compared to normative pregnancies [7].

While some interventions—such as midwife-led continuity of care models, screening for lower genital tract infections, zinc supplementation, and cervical cerclage—can reduce the likelihood of preterm birth in some subgroups of women, the majority of women who experience a preterm birth do not have a clear risk factor or causative agent [8,9]. For women who experience spontaneous preterm labour, tocolysis with an effective pharmacological agent can be used to reduce, arrest, or slow uterine contractions [10], thereby delaying birth [1]. Several drugs (such as betamimetics, calcium channel blockers, and oxytocin receptor antagonists) can delay birth by 2–7 days. Although trial evidence suggests that these drugs do not have any independent benefits in terms of improving substantive perinatal health outcomes [11,12,13], they can act a temporizing measure to administer antenatal corticosteroids to accelerate foetal lung maturity or allow a woman in preterm labour to be transferred to a higher level of care [14].

Several Cochrane systematic reviews have been published on the effects of different tocolytics on maternal and newborn outcomes [11,12,13]. These Cochrane reviews included randomized trials only and generally concluded that there is insufficient evidence regarding the benefits and possible harms of tocolysis in certain subpopulations of women. These include women experiencing extreme prematurity (i.e., birth < 28 weeks’ gestation), which affects approximately 4% of all women; women with multiple pregnancies (1–2% of all pregnant women); and women with a growth-restricted foetus, which affects nearly 20% of pregnant women in some low- and middle-income countries (LMICs) [15,16].

To address this knowledge gap, Miyazaki et al. conducted a systematic review in 2016 that aimed to identify both randomized and non-randomized studies on the use of tocolysis among women with extremely preterm birth, multiple gestations, and growth-restricted babies [17]. The review identified only seven studies (three trials and four observational studies) on women experiencing extremely preterm birth but no studies for women with multiple gestation or growth-restricted babies. In light of this limited evidence base, the authors concluded that the effectiveness of tocolysis in these subpopulations of women remained unclear. As previously discussed, it is important to clarify the effectiveness and harmful influence of tocolytic agents. Although the former review addressed a current question and is cited frequently, its evidence is insufficient [17]. Additionally, *The Cochrane Handbook*, Chapter IV, recommends updating the review in such cases [18].

One of core functions of the WHO is the production of evidence-based guidelines to guide clinical practice, including recommendations on the use of tertiary prevention interventions for improving preterm newborn outcomes [14]. As part of their maternal and perinatal health “living guidelines” program [19], recommendations pertaining to the use of antenatal corticosteroids and tocolytic agents were prioritized for update in light of new, potentially important evidence on these interventions. Those recommendations were based on the results of studies published through 2015, and it is necessary to update and consider the influence of the results of new studies in potentially overturning the conclusions of an existing review. As part of the WHO recommendation updates, we aimed to compile and analyse all available evidence on the benefits and possible harms of using tocolysis for preterm labour management in the context of pregnant women with extremely preterm birth, multiple gestations, or growth-restricted foetuses.

## 2. Materials and Methods

### 2.1. Study Design

This systematic review and meta-analysis was performed in accordance with *The Cochrane Handbook* [20]. The review protocol was registered on the international prospective register of systematic reviews, PROSPERO (https://www.crd.york.ac.uk/prospero/, accessed on 22 January 2023) (CRD42021275269), and reported according to the Preferred Reporting Items for Systematic Reviews and Meta-Analyses (PRISMA) checklist [21] (File S1). As a systematic review of published studies, ethical approval was not required.

In this review, we had three specific clinical questions (CQ) structured using the population (P), intervention (I), comparison (C) and outcome (O) format:

- CQ1: For women who are experiencing extremely preterm, spontaneous labour (P), what is the effect of using tocolysis (I) compared to placebo or no treatment (C) on maternal and newborn outcomes (O)?
- CQ2: For women who have a multiple pregnancy and are experiencing spontaneous preterm labour (P), what is the effect of using tocolysis (I) compared to placebo or no treatment (CO) on maternal and newborn outcomes (O)?
- CQ3: For women who have a growth-restricted foetus and are experiencing spontaneous preterm labour (P), what is the effect of using tocolysis (I) compared to placebo or no treatment (CO) on maternal and newborn outcomes (O)?

### 2.2. Eligibility Criteria

#### 2.2.1. Population

We pre-specified inclusion criteria for the subpopulations of women, identified in the clinical questions, in whom the effectiveness of tocolytics was uncertain (women in preterm labour and extremely preterm birth, experiencing a multiple pregnancy, or with a growth-restricted foetus, as defined by the study authors). Extremely preterm birth was defined as birth before 28 weeks of gestation. Multiple pregnancies refer to the development of more than one foetus concurrently. Intrauterine growth restriction (IUGR) is characterized by a deviation or reduction from the expected foetal growth pattern, taking into account the growth potential of a specific infant based on foetus race and gender [22]. For all questions, we included studies that involved any pregnant women receiving tocolysis for preterm labour as long as stratified data were reported for our specific sub-populations of interest (i.e., women experiencing preterm birth less than 28 weeks of gestation; pregnant women with multiple pregnancies; and pregnant women with growth-restricted foetuses). 

#### 2.2.2. Intervention and Comparison

Studies were included if they used any tocolytic agent—such as calcium channel blockers, betamimetics, oxytocin receptor antagonists, or other known tocolytic agents—compared with placebo or no treatment. Studies were included if tocolysis was given in a single administration or in combination with other tocolytic agents. 

#### 2.2.3. Outcomes

Maternal and newborn outcomes were pre-specified. 

Primary outcomes:

Maternal outcomes:

- Maternal death;
- Maternal infection—chorioamnionitis or endometritis;
- Cessation of treatment due to adverse drug reaction.

Newborn outcomes:

- Perinatal death;
- Preterm birth (<28 weeks, <32 weeks, <34 weeks, and <37 weeks);
- Delay in birth (48 h, 7 days).

Secondary outcomes:

Maternal outcomes:

- Caesarean section;
- Adverse effects (e.g., tachycardia, hypotension, palpitations, shortness of breath, chest pain, pulmonary oedema, hypokalaemia, and hyperglycaemia).

Newborn outcomes:

- Interval between trial entry and birth;
- Gestational age at birth;
- Foetal death;
- Neonatal death—up to 7 days, up to 28 days;
- Infant death;
- Respiratory morbidity: respiratory distress syndrome (RDS), chronic lung disease, persistent pulmonary hypertension of the newborn, and mechanical ventilation.
- Gastrointestinal morbidity: necrotising enterocolitis;
- Neonatal infection;
- Neurodevelopmental morbidity: intraventricular haemorrhage (IVH), infant long-term neurological development (psychomotor, mental), and cerebral palsy;
- Birth weight: mean birth weight;
- Birth weight <2000 g, <2500 g;
- Admission to the NICU;
- Parent ductus arteriosus;
- Premature closure of the ductus arteriosus;
- Foetal tachycardia;
- Foetal hypoglycaemia.

#### 2.2.4. Types of Studies

We deemed both randomized and non-randomized studies to be admissible, provided that they tackled one of the three pertinent clinical questions of interest. Individual, cluster, or quasi-randomized controlled trials (RCTs) were acceptable, as were non-randomized studies with control groups, such as controlled before–after studies, prospective or retrospective cohort studies, and case-control studies. Head-to-head studies (i.e., those that did not utilize a placebo or no treatment as a comparator) were excluded. Studies were eligible regardless of language, setting, or year of publication.

### 2.3. Literature Search

We searched five bibliographic databases from 10 to 15 July 2021—MEDLINE (Ovid), Embase (embase.com), the Cochrane Central Register of Controlled Trials (CENTRAL) (Wiley), CINAHL (EBSCO host), and the WHO Global Index Medicus databases—using a combination of index terms and text words related to the review concept. Below, we present the search strategy for Clinical Questions 1 and 3 as applied to the MEDLINE (Ovid) database. The comprehensive search strategies are available in Table S1. As this review is an update of the systematic review by Miyazaki et al., the five databases and updated search terms were based on that earlier review. As the Miyazaki et al. review used a search date of February 2014, we searched for new studies published between February 2014 and July 2021. We also searched the reference lists of retrieved studies for additional potentially eligible studies. Duplicates were removed manually.

#### Search Strategy for CQ1 and CQ3, MEDLINE (Ovid)

1. exp *Tocolytic Agents/ad, tu
2. exp *Tocolytic Agents/and (ci or de of dt).fs.
*Tocolysis/
4. exp Tocolytic Agents/ae, po, to
5. Tocolysis/ae
6. or/1–5
7. exp *Obstetric Labor, Premature/pc
8. exp Foetus Development/
9. exp Birth Weight/
10. exp Infant, Low Birth Weight/
11. or/7–10
12. 6 and 11
13. or/4–5
14. exp Foetus/
15. Obstetric Labor Complications/
16. Pregnancy, Prolonged/
17. exp Pregnancy Outcome/
18. Foetus Death/
19. Maternal Death/
20. exp Infant, Newborn/
21. Prenatal Exposure Delayed Effects/
22. or/14–21
23. 13 and 22
24. or/12, 23
25. limit 24 to humans
26. limit 25 to (biography or case reports or comment or congress or consensus development conference or consensus development conference, nih or editorial or guideline or historical article or interactive tutorial or interview or introductory journal article or lecture or news or newspaper article or overall or patient education handout or practice guideline or “review” or “scientific integrity review” or systematic review)
27. limit 26 to meta analysis
28. 26 not 27
29. 25 not 28
30. (tocoly* or Albuterol or Fenoterol or Hexoprenaline or Indomethacin or Isoxsuprine or Magnesium Sulfate or Nifedipine or Nylidrin or Ritodrine or Terbutaline).mp.
31. ((((foetus or foetus or baby or babies or birth or infant* or neonate* or newborn* or labor or labour) adj2 (development or growth or matur* or weight or prematur* or preterm)) or (gestation* adj2 (age or period))) not (“patent ductus arteriosus” or rat* or animal*)).mp.
32. (growth adj3 restrict*).mp.
33. or/31–32
34. 30 and 33
35. MEDLINE.st.
36. 34 not 35
37. (biograph* or case report* or comment or congress* or conference* or editor* or tutorial* or interview* or lecture* or news* or handout* or guideline* or (review* not (meta analys* or metaanalys*))).mp.
38. 36 not 37
39. or/29, 38
*Ductus Arteriosus, Patent/
41. 39 not 40
42. remove duplicates from 41

### 2.4. Study Selection and Data Extraction

Two review authors (N.Y. and H.S.) screened all identified citations and extracted data independently. We resolved any discrepancies through discussion or, if required, in consultation with a third author (E.O.). We extracted the data, the first author’s name, the publication year, countries, settings, participants, interventions, comparison, study design, and outcomes. These data were extracted using a pre-designed data extraction form that was developed for this review.

### 2.5. Risk of Bias Assessment

Two reviewers evaluated the potential for bias in each study independently, using appropriate evaluation methods. The Cochrane Risk of Bias tool 1.0 was employed for randomized trials [23] and the Risk of Bias Assessment Tool for Non-randomized Studies (RoBANS) was employed for non-randomized studies [24]. In cases where disagreement arose, a discussion between the reviewers was initiated or a third reviewer was consulted to resolve the issue (E.O.).

### 2.6. Data Synthesis

We synthesized the data from randomized and non-randomized studies separately. We conducted a meta-analysis using Review Manager Version 5.4 (RevMan 5.4). We employed a fixed-effect model to synthesize data in situations where it was plausible to assume that the treatment effects estimated by the included studies were essentially identical. The summary of findings table and the forest plot present the results of this analysis. If clinical heterogeneity was present, we meta-analysed the available data using a random-effects model. When necessary, we contacted the study authors for additional information. The results were presented as risk ratios (RR) for randomized trials and odds ratios (OR) for observational studies with 95% confidence intervals (Cis). Statistical heterogeneity for meta-analyses was assessed using Chi^2^ and I^2^ statistics. We regarded heterogeneity as substantial if the I^2^ was >60% and the *p*-value was <0.10 according to the Chi^2^ test [25]. We assessed the certainty of evidence for the primary outcomes perinatal death, preterm birth < 28 weeks, preterm birth < 32 weeks, preterm birth < 34 weeks, a delay in birth ≥ 48 h, and a delay in birth ≥ 7 days using the Grading of Recommendations, Assessment, Development and Evaluation (GRADE) approach [26]. Although we planned to conduct subgroup analyses to explore differential effects of different tocolytic agents, it was not possible to conduct this as there were few studies identified.

## 3. Results

**Figure 1:**
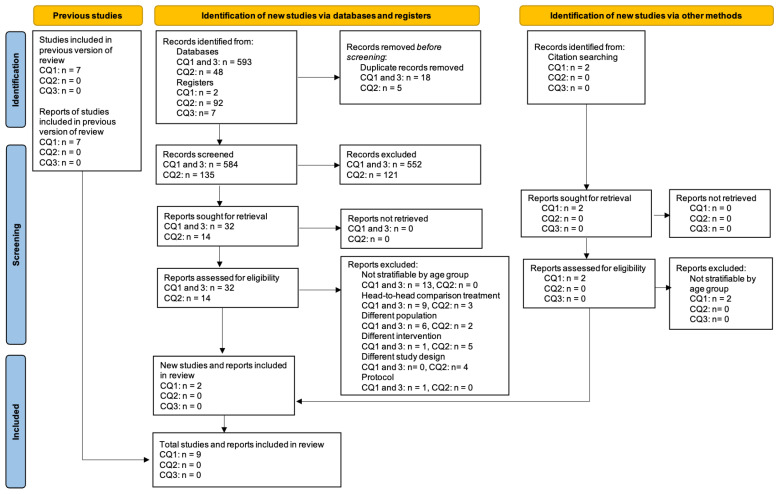
PRISMA flow diagram for review questions Q1–Q3.

The searching and screening process is shown in Figure 1. We identified 742 citations in the database search and an additional 2 studies from hand-searching. After the full text screening, a total of nine studies met the eligibility criteria and were included. Nine studies pertained to CQ1 and no studies were identified for CQ2 and CQ3. The characteristics of the included studies are reported in Table 1 (CQ1).

### Extremely Preterm Birth (CQ1)

We included three randomized trials [27,28,29] and six retrospective cohort studies [30,31,32,33,34,35] for CQ1. For CQ1, data from three trials comprised 268 women: these studies were conducted in three countries, including the United States of America (USA), Canada, and Germany [27,28,29]. Women with threatened preterm labour prior to 28 weeks of gestation were recruited into these trials due to uterine contractions and/or cervical changes. Among the trials, two trials used atosiban in the treatment arm [28,29] and one used ritodrine [27]. One trial included women between 18 and 35 weeks of gestation [28]. Two trials included women over 20 weeks of gestation and reported results for women prior to 28 weeks of gestation separately [27,29]. Two trials were assessed as low risk of bias in most domains. In contrast [27,29], one trial was assessed as unclear in most domains and at a high risk of performance bias [28].

The six cohort studies included 5590 women in the USA (5 studies) [30,31,32,34,35] and Canada (1 study) [33]. These studies assessed the effects of tocolytic agents, including indomethacin (4 cohort studies) [25,26,27,29], magnesium sulfate (MgSO_4_, 1 cohort) [33], and various types of tocolytic medications (i.e., magnesium sulfate, indomethacin, and nifedipine, used singly or in combination; 1 cohort study). Shalabi 2017 included infants who received magnesium sulfate for any indication; we were unable to obtain further clarification. The six cohort studies included women or infants born between 14 weeks and 29 weeks of gestation [30,31,32,33,34,35]; two cohort studies involved women over 14 weeks of gestation [30,34]. We rated two cohort studies as having a low risk of bias in most domains [30,33]. Two other studies were assessed as having a high risk of bias due to a lack of adjustment for potential confounding variables [31,35], and one study was assessed as having a high risk of bias due to selective outcome reporting (side effects and adverse outcomes were not reported) [34]. The six cohort studies were assessed as having an unclear risk of bias in most domains [32].

#### Maternal, Foetal, and Neonatal Outcomes in Extremely Preterm Birth

We analysed the primary outcomes using the three trials and three cohort studies and summarized the results in a summary of findings table (Table 2). No maternal outcomes were reported.

**Figure 2:**
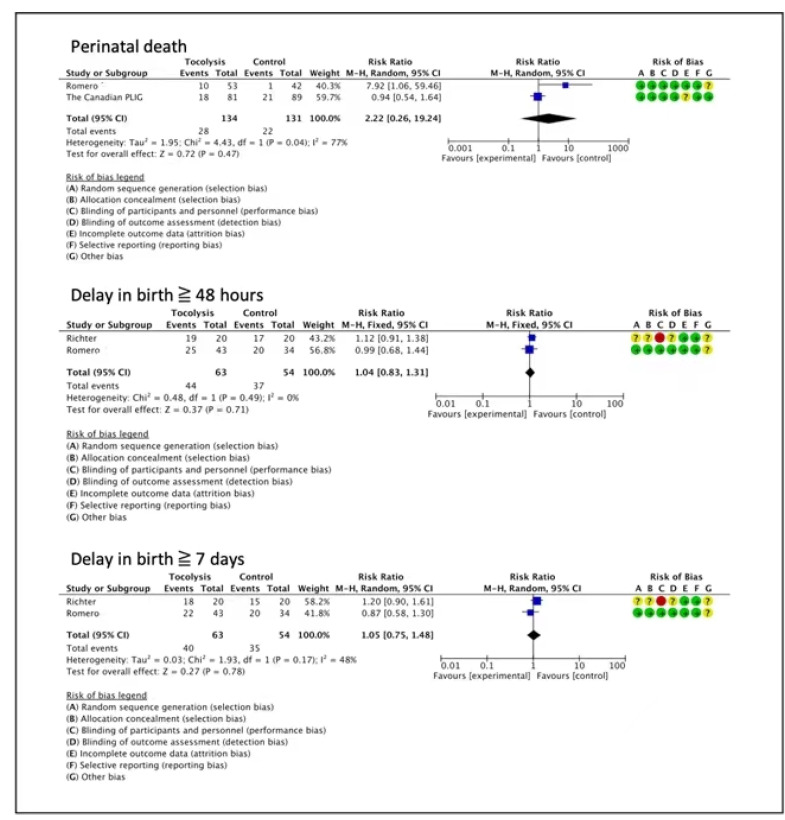
Tocolytics compared to placebo or no treatment—extreme prematurity (RCTs) [27,28,29].

Perinatal death was reported in two trials, and the intervention group did not exhibit a clear difference compared to placebo or no treatment (RR 2.22, 95% CI 0.26 to 19.24, 2 trials, 265 neonates, very low certainty evidence) (Figure 2) [27,29]. As is shown by the meta-analysis of data from two trials, there may not be a difference in a delay in birth ≥ 48 h (RR 1.04, 95% CI 0.83 to 1.31, two trials, 117 women, low-certainty of evidence) [28,29], and a delay in birth ≥ 7 days (RR 1.05, 95% CI 0.75 to 1.48, two trials, 117 women, low-certainty evidence) (Figure 2) [28,29].

**Figure 3:**
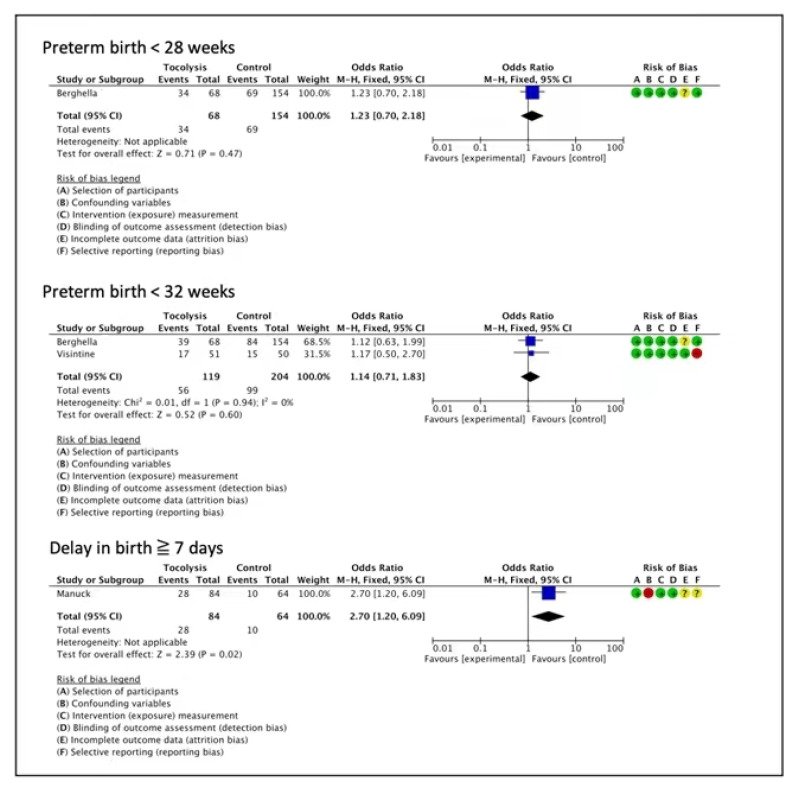
Tocolytics compared to placebo or no treatment—extreme prematurity (observational studies) [30,34,35].

In the non-randomized evidence, a cohort study suggested that tocolysis provided benefits for delaying birth for 7 days, although the certainty of evidence was very low (OR 2.70, 95%CI 1.20 to 6.09, one study, 148 women) (Figure 3) [35]. One cohort study reported no clear difference for preterm birth < 28 weeks (OR 1.23, 95% CI 0.70 to 2.18, one study, 222 women, very low certainty of evidence) [30], and two cohort studies indicated no effects on preterm birth < 32 weeks (OR 1.14, 95% CI 0.71 to 1.83, two studies, 323 women, very low certainty of evidence) [30,34], (Figure 3).

For the review’s secondary outcomes, data were available from four cohort studies (see forest plots for each outcome in Figure S1). In three cohort studies, tocolysis appeared to possibly reduce the incidence of neonatal death (OR 0.69, 95% CI 0.60 to 0.80, three studies, 4725 neonates, low-certainty evidence) [30,33,35]. One cohort study showed that tocolysis may reduce the incidence of IVH Grade Three or above or periventricular leukomalacia (OR 0.79, 95% CI 0.69 to 0.92, one study, 4141 neonates, low-certainty evidence) and the use of mechanical ventilation (OR 0.65, 95% CI 0.57 to 0.75, one study, 4355 neonates, low-certainty evidence), though the incidence of nosocomial infection may increase (OR 1.15, 95% CI 1.01 to 1.31, one study, 4355 neonates, low-certainty evidence) [33]. There were no clear differences for gestational age at birth (mean difference (MD) 0.90, 95% CI −0.10 to 1.90, one study, 50 neonates, very low-certainty evidence) [31], bronchopulmonary dysplasia (OR 0.93, 95% CI 0.81 to 1.06, two studies, 3692 neonates, low-certainty evidence) [33,35], necrotizing enterocolitis (NEC) (OR 1.03, 95% CI 0.85 to 1.26, three studies, 4626 neonates, very low-certainty evidence) [27,28,30], IVH (OR 1.15, 95% CI 0.69 to 1.94, two studies, 286 neonates, very low-certainty evidence) [32,35], patent ductus arteriosus (PDA) (OR 2.00, 95% CI 0.87 to 4.61, one study, 138 neonates, very low-certainty evidence) [32], retinopathy of prematurity (Stage 3 or above or treated) (OR 0.84, 95% CI 0.69 to 1.03, one study, 3017 neonates, very low-certainty evidence) [33], acute kidney injury (AKI) at 7 days (OR 0.84, 95% CI 0.14 to 5.16, one study, 50 neonates, very low-certainty evidence), AKI at 7–30 days (OR 1.63, 95% CI 0.43 to 6.17, one study, 50 neonates, very low-certainty evidence) [31], spontaneous intestinal perforations, (OR 1.07, 95% CI 0.78 to 1.47, two studies, 4440 neonates, very low-certainty evidence) [32,33], and mean birth weight (MD −12.59, 95% CI −25.47 to 0.29, two studies, 4405 neonates, very low-certainty evidence) [31,33].

## 4. Discussion

This systematic review and meta-analysis evaluated the effects of tocolysis in women with extremely preterm birth, multiple gestations, and growth-restricted foetuses. We identified nine studies on women with extremely preterm birth, though there was very low certainty evidence for most outcomes. While tocolytics appear to have delayed preterm birth in these women, we were unable to draw firm conclusions on the benefits and possible harms for women or newborns. There were no studies on tocolytic use in women with multiple gestations or growth-restricted foetuses.

We did not find any randomized trials that assessed tocolytic agents in women with extremely preterm labour other than those included in the previous iteration of this review [17]. We identified two new cohort studies that were added to the four cohort studies previously identified [17] for analysis. The meta-analysis of these trials showed that there were no clear differences in perinatal death and delays in birth of ≥ 48 h and ≥7 days between the tocolysis and placebo groups. In contrast, the meta-analysis of the cohort studies showed that tocolysis might increase the delay in birth by 7 days or more. One cohort study showed that tocolysis might increase the incidence of nosocomial infection and reduce the incidence of neonatal death, IVH Grade Three or above or periventricular leukomalacia, and the use of mechanical ventilation. For other outcomes assessing the effects of the tocolytic agents, the results did not demonstrate differences between groups. The studies included for women with extremely preterm births did not report on maternal outcomes aside from the caesarean section rate, in which there was no clear difference.

In their 2015 guidelines, the WHO did not recommend the routine use of tocolytic treatments (acute and maintenance treatments) for women at risk of imminent preterm birth to improve newborn outcomes [14]. While some studies suggest that tocolysis may benefit pregnant women and their infants, there is a lack of consensus on whether and how tocolysis should be used to improve preterm birth outcomes [10]. Limited evidence from Cochrane systematic reviews shows that tocolytic agents—including betamimetics, calcium channel blockers, MgSO_4_, oxytocin receptor antagonists, cyclo-oxygenase, ethanol, and nitric oxide donors—do not appear to have independent benefits in terms of improving substantive perinatal health outcomes, though some options do delay birth [11,12,13,36,37,38,39]. However, the latest Cochrane systematic review evidence, which used a network meta-analysis, showed that six tocolytic drug classes (betamimetics, calcium channel blockers, magnesium sulphate, oxytocin receptor antagonists, and nitric oxide donors) and their combinations were probably or possibly effective in delaying preterm birth for 48 h and 7 days [40]. Delaying preterm birth can allow time for important, globally recommended interventions to improve newborn outcomes, such as the administration of antenatal corticosteroids or transfer to higher level care [14].

In the current, contemporary meta-analysis, we identified no definitive evidence as to whether tocolytic treatments are effective or safe for women giving birth at <28 weeks of pregnancy. While some outcomes were suggestive of benefits, the certainty of the evidence was assessed as low to very low as these estimates were derived from small sample sizes and thus must be interpreted with considerable caution. It remains unclear whether using tocolytic drugs improves preterm birth outcomes in women at <28 weeks of pregnancy. There is no evidence available to assess the potential benefits and harms of tocolytic therapies for women with multiple gestations and growth-restricted foetuses. Further trials are needed before specific conclusions can be drawn on the use of tocolytic therapy for delaying preterm birth and improving preterm birth outcomes in these populations.

The strengths of this review include its adherence to the standards of *The Cochrane Handbook* in order to ensure that all potential biases were identified and addressed. At least two review authors carried out the screening and data extraction, and assessed the risk of bias independently. The findings of this study increase our understanding of the effects of tocolytic therapy on women with pregnancies < 28 weeks of gestation. However, the review has some limitations. First, the results of the meta-analysis were from limited data; thus, it is likely that conclusions may change when additional evidence comes to light in the future. Second, we included one study that included infants who received magnesium sulfate for any indication. It is possible that the magnesium sulfate was used for indications such as pre-eclampsia or foetal neuroprotection.

Further well-designed studies are needed to evaluate the effects and possible harms of tocolytic agents to improve preterm birth outcomes, particularly for women with multiple gestations or with growth-restricted foetuses. The range of maternal and newborn mortality and morbidity outcomes, as well as maternal outcomes (e.g., maternal infections, cessation of treatment due to adverse drug reaction) and harms (e.g., tachycardia, hypotension, and chest pain) should be reported to obtain meaningful data for clinical decision-making. Acknowledging that randomized trials involving these women can be challenging to implement, well-conducted retrospective observational studies would nonetheless provide useful evidence to guide clinical decision-making internationally.

## 5. Conclusions

The current body of evidence is inadequate to establish the impact of tocolytics on women who have less than 28 weeks of gestation, women who are carrying multiple pregnancies, and women who have a growth-restricted foetus. Further well-designed studies, including randomised controlled trials and retrospective observational studies, are needed to evaluate the effects and possible harms of tocolytic agents to improve preterm birth outcomes.

## Figures and Tables

**Table 1:** Characteristics of included studies for CQ1: women experiencing extremely preterm birth (<28 weeks of gestation).

Author/Year	Country	Participants	Intervention	Control	Study Design
The Canadian PLIG, 1992 [27]	Canada	Women between 20 and 35 weeks of gestation with uterine contractions at a rate of four per 20 min or six per 60 min or any uterine activity with either rupture of membranes or cervical dilatation by 2 cm or more (N = 708 women; N = 151 women at <28 weeks).	Ritodrine (n = 352 women; 76 women at <28 weeks) (Intravenous infusion of ritodrine in 5% dextrose at a rate of 0.35 mg/min until the cessation of uterine activity, the failure of therapy, or the occurrence of impermissible maternal side effects).	Placebo (n = 356 women; 75 women at <28 weeks) (Dextrose solution alone without ritodrine)	RCT
Richter, 2005 [28]	Germany	Women between 18 and 24 weeks of gestation and with a uterine contraction duration of >30 s and rate ≥ 4/30 min. Cervical effacement > 50% and cervical dilatation of 0–3 cm (nulliparous) or 1–3 cm (primiparous and multiparous) (N = 40 women).	Atosiban (n = 20 women) (Initial intravenous infusion of 6.75 mg of atosiban in 0.9 mL of sodium chloride, followed by high dosage of infusion (300 lg/min) for 3 h and then low dosage (100 lg/min) up to 45 h).	Placebo (n = 20 women)	RCT
Romero, 2000 [29]	USA	Women between 20 and 33 weeks of gestation with intact membranes, cervical dilatation of 1 to ≤3 cm, who were in preterm labor (requiring the presence of ≥4 uterine contractions over 30 min, each lasting at least 40 s) (N = 501 women; N = 77 women at <28 weeks).	Atosiban (n = 250 women; 43 women at <28 weeks) (Initial intravenous infusion of 6.75 mg of atosiban over 1 min and followed by an infusion of 300 μg/min of atosiban for 3 h, and then 100 μg/min for up to 45 h).	Placebo (n = 251 women; 34 women at <28 weeks) (Matching placebo contained the same formulation without the 5% mannitol solution of atosiban).	RCT
Berghella, 2009 [30]	USA	Women between 14 and 25 weeks of gestation with suspected cervical dilation ≥ 1 cm (N = 222 women).	Indomethacin plus some with options (e.g., physical-exam-indicated cerclage, antibiotics, amniocentesis, and Trendelenberg positioning) (n = 68 women) (50 mg oral indomethacin, followed by 25 mg orally every 6 h for a maximum of 48 h).	No treatment, plus some with cerclage (n = 154 women).	Observational study (cohort study)
Brichta, 2021 [31]	USA	Preterm neonates born <29 weeks of gestation at a level III neonatal intensive care unit (NICU); Women in extremely preterm labor were given a loading dose of indomethacin, followed by 48 to 72 h of maintenance dosing (N = 55 women).	Indomethacin-exposed neonates (n = 17); 15 women received between 3 and 31 doses of indomethacin (9.8 ± 6.4; mean ± SDs). Most of the women (53%) received their initial doses of indomethacin between days 1–6 prior to delivery, but 40% of indomethacin-exposed neonates had received maternal indomethacin over one week prior to birth.	Indomethacin-unexposed neonates (n = 38).	Observational study (cohort study)
Cape, 2010 [32]	USA	All women <29 weeks of gestation with threatened premature rupture of membranes (N = 138 women).	Indomethacin (n = 69 infants) Neonates <29 weeks of gestation exposed to in utero indomethacin.	Indomethacin-unexposed infants (n = 69).	Observational study (cohort study)
Shalabi, 2017 [33]	Canada	Infants born between 22 and 27 weeks of gestation; (N = 4926 infants) Infants with a major congenital anomaly or who were moribund on admission were excluded.	Infants who received intrapartum magnesium sulfate (MgSO_4_) for any indication (n = 2300 infants).	Intrapartum MgSO_4_-unexposed Infants (n = 2055).	Observational study (cohort study)
Visintine, 2008 [34]	USA	Asymptomatic women followed from 14 weeks through 23 weeks 6 days gestation with a short cervical length, defined as <25 mm, who had an ultrasound-indicated guided cerclage placed (N = 101 women).	Indomethacin plus cerclage (50 mg initially orally or rectally, followed by 25 mg orally every 6 h for approximately 48 h.) (n = 51 women).	Cerclage only (n = 50 women).	Observational study (cohort study)
Manuck, 2012 [35]	USA	Women with a singleton non-anomalous foetus in spontaneous preterm labour with intact membranes, between 20 and 23.9 weeks of gestation, and with cervical dilation ≥ 1 cm and effaced > 50% (N = 148 women).	Tocolytic medication (database record of tocolytic treatment used, i.e., magnesium sulfate, indomethacin, or nifedipine, either used singly or in combination) (n = 84 women).	No treatment (n = 64 women).	Observational study (cohort study)

RCT: randomized controlled trial, USA: United States of America.

**Table 2:** Summary of findings table on tocolysis for women with extremely preterm birth.

**Tocolytics compared to placebo or no treatment—extreme pretermaturity_Randomized controlled trials**					
**Patient or population: preterm birth; Setting: USA, Canada, Germany; Intervention: tocolysis; Comparison: placebo**					
**Outcomes**	Anticipated absolute effects * (95% CI)		Relative effect (95% CI)	Number of participants (studies)	Certainty of the evidence (GRADE)
	Risk with placebo	Risk with Tocolysis			
Perinatal death	168 per 1000	373 per 1000 (44 to 1000)	RR 2.22 (0.26 to 19.24)	265 (Two RCTs)	⨁◯◯◯ Very low ^a,b^
Delay in birth ≥ 48 h	685 per 1000	713 per 1000 (569 to 898)	RR 1.04 (0.83 to 1.31)	117 (Two RCTs)	⨁⨁◯◯ Low ^b^
Delay in birth ≥ 7 days	648 per 1000	681 per 1000 (486 to 959)	RR 1.05 (0.75 to 1.48)	117 (wo RCTs)	⨁⨁◯◯ Low ^b^
**Tocolytics compared to placebo or no treatment—extreme pretermaturity_cohort studies**					
**Patient or population: preterm birth; Setting: USA, Canada; Intervention: tocolysis; Comparison: placebo or no treatment**					
Preterm birth < 28 weeks	448 per 1000	500 per 1000 (362 to 639)	OR 1.23 (0.70 to 2.18)	222 (One observational study)	⨁◯◯◯ Very low ^b^
Preterm birth < 32 weeks	485 per 1000	518 per 1000 (401 to 633)	OR 1.14 (0.71 to 1.83)	323 (Two observational studies)	⨁◯◯◯ Very low ^c^
Delay in birth ≥ 7 days	156 per 1000	333 per 1000 (182 to 530)	OR 2.70 (1.20 to 6.09)	148 (One observational study)	⨁◯◯◯ Very low ^d,e^
* The risk in the intervention group (and its 95% confidence interval) is based on the assumed risk in the comparison group and the relative effect of the intervention (and its 95% CI). USA: United States of America; CI: confidence interval; MD: mean difference; OR: odds ratio; RR: risk ratio					
GRADE Working Group grades of evidence Low certainty: our confidence in the effect estimate is limited: the true effect may be substantially different from the estimate of the effect. Very low certainty: we have very little confidence in the effect estimate: the true effect is likely to be substantially different from the estimate of effect.					

Explanations: ^a^ Statistical heterogeneity (l2 ≥ 60%). ^b^ The estimation was based on a small sample size. The confidence intervals were broad and intersected the line denoting the null effect. ^c^ The confidence intervals were extensive and overlapped the line denoting the null effect. ^d^ All the studies incorporated in this analysis had some methodological constraints. ^e^ The estimation was based on the data extracted from the study with limited sample size and a minimal number of events.

## Data Availability

Data are contained within the article.

## Supplementary Materials

The following supporting information can be downloaded at: https://www.mdpi.com/article/10.3390/children10030443/s1, File S1: Preferred reporting items for systematic reviews and meta-analyses (PRISMA) checklist; Figure S1: Forest plots of secondary outcomes for tocolysis amongst women with extreme prematurity (<28 weeks’ gestation) (CQ1); Table S1: Search strategies.
