# Supplementary material for: Tocolytic Therapy Inhibiting Preterm Birth in High-Risk Populations: A Systematic Review and Meta-Analysis
Source: Children (Basel). 2023 Feb 24;10(3):443. doi: 10.3390/children10030443 (PMC10047044; doi:10.3390/children10030443)
Supplement: Supplementary file 1 [file children-10-00443-s001.zip › children-2206198-supplementary/Supplementary File(s)/Figure S1.pdf]

Neonatal death (cohort studies)

Study or Subgroup	Tocolysis Events	Tocolysis Total	Control Events	Control Total	Weight	Odds Ratio M-H, Fixed, 95% CI	Risk of Bias A	B	C	D	E	F
Berghella 2009	21	68	59	154	5.6%	0.72 [0.39, 1.32]	+	+	+	+	?	+
Manuck 2012	30	84	35	64	5.8%	0.46 [0.24, 0.89]	+	−	+	+	?	?
Shalabi 2017	333	2055	497	2300	88.6%	0.70 [0.60, 0.82]	+	+	+	+	+	?
**Total (95% CI)**		**2207**		**2518**	**100.0%**	**0.69 [0.60, 0.80]**						
Total events	384		591									

Heterogeneity: $Chi^2 = 1.49$, df = 2 (P = 0.48); $I^2 = 0\%$

Test for overall effect: Z = 5.03 (P < 0.00001)

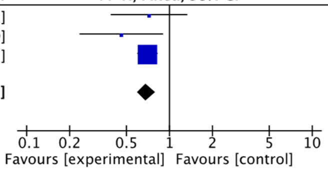

Risk of bias legend
(**A**) Selection of participants
(**B**) Confounding variables
(**C**) Intervention (exposure) measurement
(**D**) Blinding of outcome assessment (detection bias)
(**E**) Incomplete outcome data (attrition bias)
(**F**) Selective reporting (reporting bias)

Gestational age at birth (cohort study)

Study or Subgroup	Tocolysis Mean	Tocolysis SD	Tocolysis Total	Control Mean	Control SD	Control Total	Weight	Mean Difference IV, Fixed, 95% CI	Risk of Bias A	B	C	D	E	F
Brichta 2021	26.4	1.75	35	25.5	1.6	15	100.0%	0.90 [−0.10, 1.90]	+	−	?	+	+	?
**Total (95% CI)**			**35**			**15**	**100.0%**	**0.90 [−0.10, 1.90]**						

Heterogeneity: Not applicable

Test for overall effect: Z = 1.77 (P = 0.08)

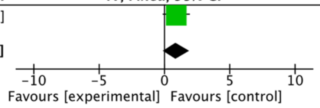

Risk of bias legend
(**A**) Selection of participants
(**B**) Confounding variables
(**C**) Intervention (exposure) measurement
(**D**) Blinding of outcome assessment (detection bias)
(**E**) Incomplete outcome data (attrition bias)
(**F**) Selective reporting (reporting bias)

Bronchopulmonary dysplasia (cohort studies)

Study or Subgroup	Tocolysis Events	Tocolysis Total	Control Events	Control Total	Weight	Odds Ratio M-H, Fixed, 95% CI	Risk of Bias A	B	C	D	E	F
Manuck 2012	13	84	4	64	0.8%	2.75 [0.85, 8.87]	+	−	+	+	?	?
Shalabi 2017	706	1732	779	1812	99.2%	0.91 [0.80, 1.04]	+	+	+	+	+	?
**Total (95% CI)**		**1816**		**1876**	**100.0%**	**0.93 [0.81, 1.06]**						
Total events	719		783									

Heterogeneity: $Chi^2 = 3.35$, df = 1 (P = 0.07); $I^2 = 70\%$

Test for overall effect: Z = 1.11 (P = 0.27)

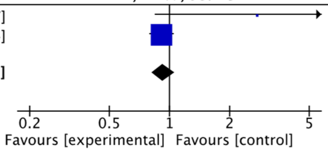

Risk of bias legend
(**A**) Selection of participants
(**B**) Confounding variables
(**C**) Intervention (exposure) measurement
(**D**) Blinding of outcome assessment (detection bias)
(**E**) Incomplete outcome data (attrition bias)
(**F**) Selective reporting (reporting bias)

Necrotising enterocolitis (cohort studies)

Study or Subgroup	Tocolysis Events	Tocolysis Total	Control Events	Control Total	Weight	Odds Ratio M-H, Fixed, 95% CI	Risk of Bias A	B	C	D	E	F
Cape 2010	13	69	13	69	5.3%	1.00 [0.43, 2.35]	?	+	?	+	?	?
Manuck 2012	2	84	1	64	0.6%	1.54 [0.14, 17.33]	+	−	+	+	?	?
Shalabi 2017	202	2045	220	2295	94.1%	1.03 [0.85, 1.26]	+	+	+	+	+	?
**Total (95% CI)**		**2198**		**2428**	**100.0%**	**1.03 [0.85, 1.26]**						
Total events	217		234									

Heterogeneity: $Chi^2 = 0.11$, df = 2 (P = 0.95); $I^2 = 0\%$

Test for overall effect: Z = 0.34 (P = 0.73)

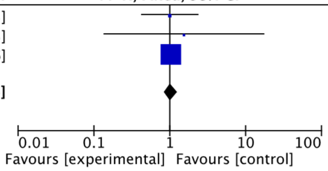

Risk of bias legend
(**A**) Selection of participants
(**B**) Confounding variables
(**C**) Intervention (exposure) measurement
(**D**) Blinding of outcome assessment (detection bias)
(**E**) Incomplete outcome data (attrition bias)
(**F**) Selective reporting (reporting bias)

Intraventricular haemorrhage (cohort studies)

Study or Subgroup	Tocolysis Events	Tocolysis Total	Control Events	Control Total	Weight	Odds Ratio M-H, Fixed, 95% CI	Risk of Bias A B C D E F
Cape 2010	31	69	34	69	70.6%	0.84 [0.43, 1.64]	? + ? + ? ?
Manuck 2012	20	84	9	64	29.4%	1.91 [0.80, 4.54]	+ - + + ? ?
**Total (95% CI)**		**153**		**133**	**100.0%**	**1.15 [0.69, 1.94]**	
Total events	51		43				

Heterogeneity: $Chi^2 = 2.17$, df = 1 (P = 0.14); $I^2 = 54\%$
Test for overall effect: Z = 0.54 (P = 0.59)

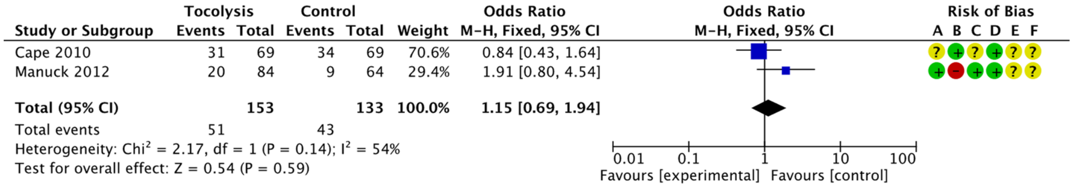

Risk of bias legend
(**A**) Selection of participants
(**B**) Confounding variables
(**C**) Intervention (exposure) measurement
(**D**) Blinding of outcome assessment (detection bias)
(**E**) Incomplete outcome data (attrition bias)
(**F**) Selective reporting (reporting bias)

Intraventricular haemorrhage grade 3 or above or periventricular leukomalacia (cohort study)

Study or Subgroup	Tocolysis Events	Tocolysis Total	Control Events	Control Total	Weight	Odds Ratio M-H, Fixed, 95% CI	Risk of Bias A B C D E F
Shalabi 2017	429	1970	564	2171	100.0%	0.79 [0.69, 0.92]	+ + + + + ?
**Total (95% CI)**		**1970**		**2171**	**100.0%**	**0.79 [0.69, 0.92]**	
Total events	429		564				

Heterogeneity: Not applicable
Test for overall effect: Z = 3.16 (P = 0.002)

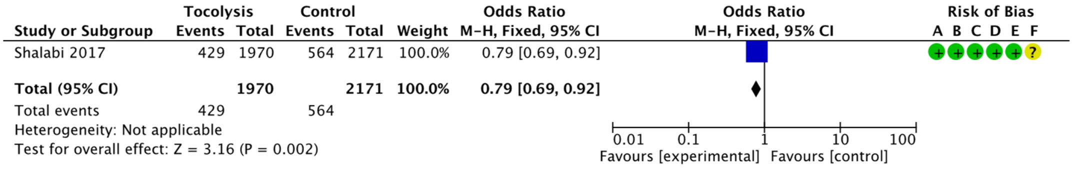

Risk of bias legend
(**A**) Selection of participants
(**B**) Confounding variables
(**C**) Intervention (exposure) measurement
(**D**) Blinding of outcome assessment (detection bias)
(**E**) Incomplete outcome data (attrition bias)
(**F**) Selective reporting (reporting bias)

Patent ductus arteriosus (cohort study)

Study or Subgroup	Tocolysis Events	Tocolysis Total	Control Events	Control Total	Weight	Odds Ratio M-H, Fixed, 95% CI	Risk of Bias A B C D E F
Cape 2010	58	69	50	69	100.0%	2.00 [0.87, 4.61]	? + ? + ? ?
**Total (95% CI)**		**69**		**69**	**100.0%**	**2.00 [0.87, 4.61]**	
Total events	58		50				

Heterogeneity: Not applicable
Test for overall effect: Z = 1.63 (P = 0.10)

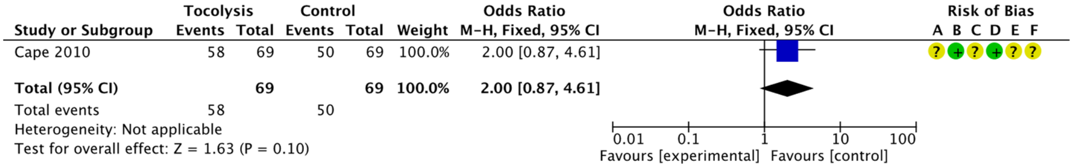

Risk of bias legend
(**A**) Selection of participants
(**B**) Confounding variables
(**C**) Intervention (exposure) measurement
(**D**) Blinding of outcome assessment (detection bias)
(**E**) Incomplete outcome data (attrition bias)
(**F**) Selective reporting (reporting bias)

Nocosomial infection (cohort study)

Study or Subgroup	Tocolysis Events	Tocolysis Total	Control Events	Control Total	Weight	Odds Ratio M-H, Fixed, 95% CI	Risk of Bias A B C D E F
Shalabi 2017	596	2055	603	2300	100.0%	1.15 [1.01, 1.31]	+ + + + + ?
**Total (95% CI)**		**2055**		**2300**	**100.0%**	**1.15 [1.01, 1.31]**	
Total events	596		603				

Heterogeneity: Not applicable
Test for overall effect: Z = 2.05 (P = 0.04)

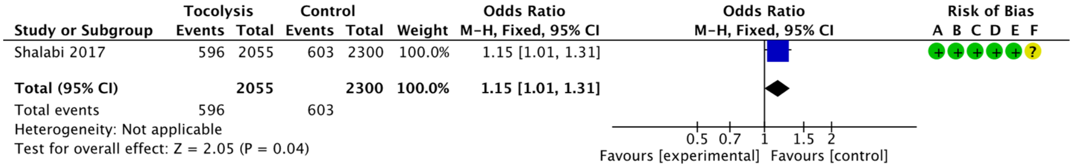

Risk of bias legend
(**A**) Selection of participants
(**B**) Confounding variables
(**C**) Intervention (exposure) measurement
(**D**) Blinding of outcome assessment (detection bias)
(**E**) Incomplete outcome data (attrition bias)
(**F**) Selective reporting (reporting bias)

Retinopathy of prematurity (stage 3 or above or treated) (cohort study)

	Tocolysis		Control			Odds Ratio	Risk of Bias					
Study or Subgroup	Events	Total	Events	Total	Weight	M-H, Fixed, 95% CI	A	B	C	D	E	F
Shalabi 2017	212	1478	255	1539	100.0%	0.84 [0.69, 1.03]	+	+	+	+	+	?
**Total (95% CI)**		**1478**		**1539**	**100.0%**	**0.84 [0.69, 1.03]**						
Total events	212		255									

Heterogeneity: Not applicable
Test for overall effect: Z = 1.69 (P = 0.09)

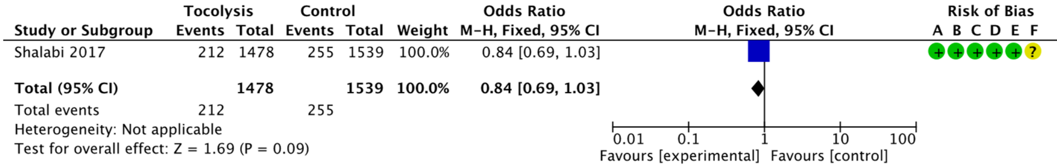

Risk of bias legend
(**A**) Selection of participants
(**B**) Confounding variables
(**C**) Intervention (exposure) measurement
(**D**) Blinding of outcome assessment (detection bias)
(**E**) Incomplete outcome data (attrition bias)
(**F**) Selective reporting (reporting bias)

Acute kidney injury (AKI) by serum creatinine (sCR) at 7 days (cohort study)

	Tocolysis		Control			Odds Ratio	Risk of Bias					
Study or Subgroup	Events	Total	Events	Total	Weight	M-H, Fixed, 95% CI	A	B	C	D	E	F
Brichta 2021	4	35	2	15	100.0%	0.84 [0.14, 5.16]	+	-	?	+	+	?
**Total (95% CI)**		**35**		**15**	**100.0%**	**0.84 [0.14, 5.16]**						
Total events	4		2									

Heterogeneity: Not applicable
Test for overall effect: Z = 0.19 (P = 0.85)

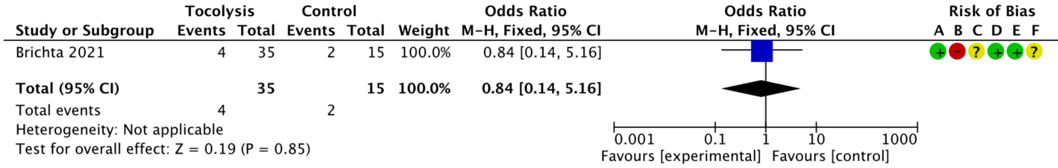

Risk of bias legend
(**A**) Selection of participants
(**B**) Confounding variables
(**C**) Intervention (exposure) measurement
(**D**) Blinding of outcome assessment (detection bias)
(**E**) Incomplete outcome data (attrition bias)
(**F**) Selective reporting (reporting bias)

Acute kidney injury (AKI) by serum creatinine (sCR) at 7-30 days (cohort study)

	Tocolysis		Control			Odds Ratio	Risk of Bias					
Study or Subgroup	Events	Total	Events	Total	Weight	M-H, Fixed, 95% CI	A	B	C	D	E	F
Brichta 2021	13	35	4	15	100.0%	1.63 [0.43, 6.17]	+	-	?	+	+	?
**Total (95% CI)**		**35**		**15**	**100.0%**	**1.63 [0.43, 6.17]**						
Total events	13		4									

Heterogeneity: Not applicable
Test for overall effect: Z = 0.71 (P = 0.48)

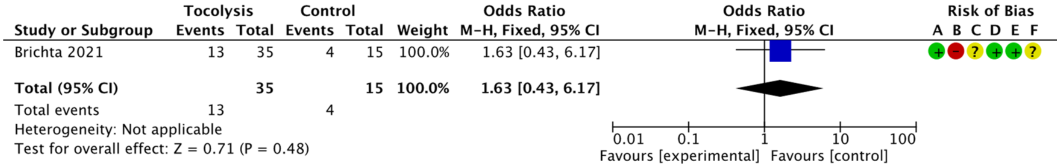

Risk of bias legend
(**A**) Selection of participants
(**B**) Confounding variables
(**C**) Intervention (exposure) measurement
(**D**) Blinding of outcome assessment (detection bias)
(**E**) Incomplete outcome data (attrition bias)
(**F**) Selective reporting (reporting bias)

Spontaneous intestinal perforations (cohort study)

	Tocolysis		Control			Odds Ratio	Risk of Bias					
Study or Subgroup	Events	Total	Events	Total	Weight	M-H, Fixed, 95% CI	A	B	C	D	E	F
Cape 2010	8	69	3	69	3.6%	2.89 [0.73, 11.37]	?	+	?	+	?	?
Shalabi 2017	69	2031	77	2271	96.4%	1.00 [0.72, 1.39]	+	+	+	+	+	?
**Total (95% CI)**		**2100**		**2340**	**100.0%**	**1.07 [0.78, 1.47]**						
Total events	77		80									

Heterogeneity: $Chi^2 = 2.16$, df = 1 (P = 0.14); $I^2 = 54\%$
Test for overall effect: Z = 0.42 (P = 0.68)

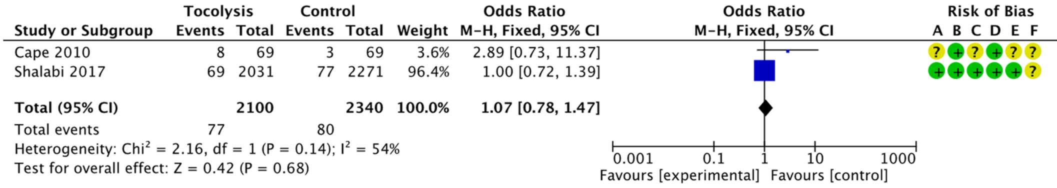

Risk of bias legend
(**A**) Selection of participants
(**B**) Confounding variables
(**C**) Intervention (exposure) measurement
(**D**) Blinding of outcome assessment (detection bias)
(**E**) Incomplete outcome data (attrition bias)
(**F**) Selective reporting (reporting bias)

Use of mechanical ventilation (cohort study)

Study or Subgroup	Tocolysis Events	Tocolysis Total	Control Events	Control Total	Weight	Odds Ratio M–H, Fixed, 95% CI	Risk of Bias A	B	C	D	E	F
Shalabi 2017	1458	2055	1816	2300	100.0%	0.65 [0.57, 0.75]	+	+	+	+	+	?
**Total (95% CI)**		**2055**		**2300**	**100.0%**	**0.65 [0.57, 0.75]**						
Total events	1458		1816									

Heterogeneity: Not applicable
Test for overall effect: Z = 6.09 (P < 0.00001)

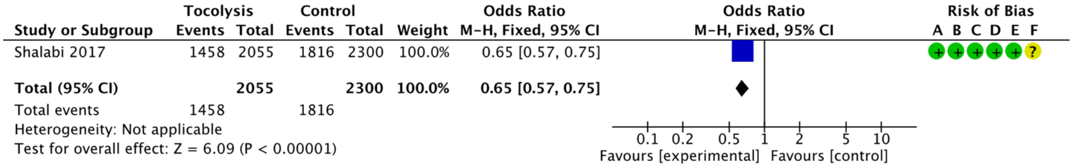

Risk of bias legend
(**A**) Selection of participants
(**B**) Confounding variables
(**C**) Intervention (exposure) measurement
(**D**) Blinding of outcome assessment (detection bias)
(**E**) Incomplete outcome data (attrition bias)
(**F**) Selective reporting (reporting bias)

Mean birth weight (cohort studies)

Study or Subgroup	Tocolysis Mean	Tocolysis SD	Tocolysis Total	Control Mean	Control SD	Control Total	Weight	Mean Difference IV, Fixed, 95% CI	Risk of Bias A	B	C	D	E	F
Brichta 2021	880	291	35	834	238	15	0.7%	46.00 [−108.27, 200.27]	+	−	?	+	+	?
Shalabi 2017	840	212	2055	853	223	2300	99.3%	−13.00 [−25.93, −0.07]	+	+	+	+	+	?
**Total (95% CI)**			**2090**			**2315**	**100.0%**	**−12.59 [−25.47, 0.29]**						

Heterogeneity: $Chi^2 = 0.56$, df = 1 (P = 0.46); $I^2 = 0\%$
Test for overall effect: Z = 1.92 (P = 0.06)

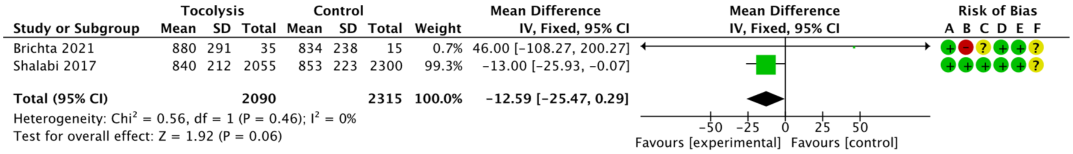

Risk of bias legend
(**A**) Selection of participants
(**B**) Confounding variables
(**C**) Intervention (exposure) measurement
(**D**) Blinding of outcome assessment (detection bias)
(**E**) Incomplete outcome data (attrition bias)
(**F**) Selective reporting (reporting bias)

Figure S1. Forest plots of secondary outcomes for tocolysis amongst women with extreme prematurity (<28 weeks' gestation) (CQ1).
