# Supplementary material for: Tocolytic Therapy Inhibiting Preterm Birth in High-Risk Populations: A Systematic Review and Meta-Analysis
Source: Children (Basel). 2023 Feb 24;10(3):443. doi: 10.3390/children10030443 (PMC10047044; doi:10.3390/children10030443)
Supplement: Supplementary file 1 [file children-10-00443-s001.zip › children-2206198-supplementary/Supplementary File(s)/Table S1.pdf]

Table S1. Search strategies.

Search strategies for CQ1 and CQ3

MEDLINE (Ovid)

ID	Query/Search terms
1	exp *Tocolytic Agents/ad, tu
2	exp *Tocolytic Agents/ and (ci or de of dt).fs.
3	*Tocolysis/
4	exp Tocolytic Agents/ae, po, to
5	Tocolysis/ae
6	or/1-5
7	exp *Obstetric Labor, Premature/pc
8	exp Fetal Development/
9	exp Birth Weight/
10	exp Infant, Low Birth Weight/
11	or/7-10
12	6 and 11
13	or/4-5
14	exp Fetus/
15	Obstetric Labor Complications/
16	Pregnancy, Prolonged/
17	exp Pregnancy Outcome/
18	Fetal Death/
19	Maternal Death/
20	exp Infant, Newborn/
21	Prenatal Exposure Delayed Effects/
22	or/14-21
23	13 and 22
24	or/12,23
25	limit 24 to humans
26	limit 25 to (biography or case reports or comment or congress or consensus development conference or consensus development conference, nih or editorial or guideline or historical article or interactive tutorial or interview or introductory journal article or lecture or news or newspaper article or overall or patient education handout or practice guideline or "review" or "scientific integrity review" or systematic review)
27	limit 26 to meta analysis
28	26 not 27
29	25 not 28
30	(tocoly* or Albuterol or Fenoterol or Hexoprenaline or Indomethacin or Isoxsuprine or Magnesium Sulfate or Nifedipine or Nylidrin or Ritodrine or Terbutaline).mp.
31	((((fetal or fetus or baby or babies or birth or infant* or neonate* or newborn* or labor or labour) adj2 (development or growth or matur* or weight or prematur* or preterm)) or (gestation* adj2 (age or period))) not ("patent ductus arteriosus" or rat* or animal*)).mp.
32	(growth adj3 restrict*).mp.
33	or/31-32
34	30 and 33
35	MEDLINE.st.
36	34 not 35

37	(biograph* or case report* or comment or congress* or conference* or editor* or tutorial* or interview* or lecture* or news* or handout* or guideline* or (review* not (meta analys* or metaanalys*))).mp.
38	36 not 37
39	or/29,38
40	*Ductus Arteriosus, Patent/
41	39 not 40
42	remove duplicates from 41

Embase (embase.com)

No.	Query
#1	'uterus spasmolytic agent'/exp/mj/dd_do,dd_cm,dd_dt,dd_ad,dd_to,dd_ct,dd_it
#2	'uterus spasmolytic agent'/exp/dd_ae
#3	'tocolysis'/mj
#4	'tocolysis'/dd_ae
#5	#1 OR #2 OR #3 OR #4
#6	'immature and premature labor'/exp/mj
#7	'parameters concerning the fetus, newborn and pregnancy'/exp
#8	'fetus development'/exp
#9	#6 OR #7 OR #8
#10	#5 AND #9
#11	#2 OR #4
#12	'labor complication'/de
#13	'fetus'/de
#14	'newborn'/de
#15	'prenatal care'/exp
#16	'fetus death'/exp
#17	'prolonged pregnancy'/de
#18	'prenatal exposure'/de
#19	#12 OR #13 OR #14 OR #15 OR #16 OR #17 OR #18
#20	#11 AND #19
#21	#10 OR #20
#22	#21 AND 'human'/de
#23	#22 AND [embase]/lim NOT [medline]/lim
#24	'editorial'/de OR 'erratum'/exp OR 'note'/de OR 'review'/de
#25	'meta analysis'/exp
#26	#24 NOT #25
#27	#23 NOT #26
#28	'case report'/exp
#29	#27 NOT #28
#30	'patent ductus arteriosus'/exp/mj
#31	#29 NOT #30

Cochrane Central Register of Controlled Trials (CENTRAL) (Wiley)

ID	Search
#1	MeSH descriptor: [Tocolytic Agents] explode all trees
#2	MeSH descriptor: [Tocolysis] explode all trees
#3	Tocoly* or Magnesium Sulfate or Ritodrine or Terbutaline or spasmoly*
#4	#1 or #2 or #3
#5	MeSH descriptor: [Obstetric Labor, Premature] explode all trees
#6	prematur* or immatur* or matur*

#7	MeSH descriptor: [Fetal Development] explode all trees
#8	MeSH descriptor: [Birth Weight] explode all trees
#9	(fetal or fetus or birth) near weight
#10	MeSH descriptor: [Fetus] explode all trees
#11	MeSH descriptor: [Obstetric Labor Complications] explode all trees
#12	MeSH descriptor: [Pregnancy, Prolonged] explode all trees
#13	MeSH descriptor: [Pregnancy Outcome] explode all trees
#14	MeSH descriptor: [Fetal Death] explode all trees
#15	MeSH descriptor: [Maternal Death] explode all trees
#16	MeSH descriptor: [Infant, Newborn] explode all trees
#17	MeSH descriptor: [Prenatal Exposure Delayed Effects] explode all trees
#18	"fetus mortality" or "fetus outcome" or "fetus risk" or "fetus mortality" or "fetus weight" or "gestational age" or "live birth" or "pregnancy outcome" or "prenatal mortality" or fetus or newborn or "labor inhibition" or "prenatal care" or "perinatal mortality" or "prenatal development" or "fetus development" or "prenatal exposure"
#19	restrict* near growth*
#20	#5 or #6 or #7 or #8 or #9 or #10 or #11 or #12 or #13 or #14 or #15 or #16 or #17 or #18 or #19
#21	#4 and #20
#22	handsrch
#23	#21 and #22

CINAHL (EBSCO host)

ID	Search Terms
S1	(MM "Tocolytic Agents+/AD/DE/TU")
S2	(MH "Tocolytic Agents+/PO/AE")
S3	S1 or S2
S4	(MH "Labor, Premature")
S5	(MH "Fetal Development+")
S6	(MH "Birth Weight")
S7	(MH "Infant, Low Birth Weight+")
S8	S4 or S5 or S6 or S7
S9	S3 and S8
S10	(MH "Fetus+")
S11	(MH "Labor Complications")
S12	(MH "Pregnancy, Prolonged")
S13	(MH "Pregnancy Outcomes")
S14	(MH "Perinatal Death")
S15	(MH "Maternal Mortality")
S16	(MH "Infant, Newborn+")
S17	(MH "Prenatal Exposure Delayed Effects")
S18	S10 or S11 or S12 or S13 or S14 or S15 or S16 or S17
S19	S2 and S18
S20	S9 or S19
S21	S20 Limiters - Exclude MEDLINE records

WHO Global Index Medicus (Global Index Medicus)

ID	Query/Search terms
1	(tocoly* OR indomethacin* OR "Magnesium Sulfate" OR Ritodrine OR Terbutaline OR spasmoly*) AND (labor OR labour OR premature OR immature OR matur*) NOT (restrict* AND growth)

Search strategies for CQ2

MEDLINE (Ovid)

ID	Query/Search terms
1	exp *Tocolytic Agents/ad, tu
2	exp *Tocolytic Agents/ and (ci or de of dt).fs.
3	*Tocolysis/
4	exp Tocolytic Agents/ae, po, to
5	Tocolysis/ae
6	or/1-5
7	exp Pregnancy, Multiple/
8	exp Multiple Birth Offspring/
9	or/7-8
10	6 and 9
11	limit 10 to humans
12	limit 11 to (biography or case reports or comment or congress or consensus development conference or consensus development conference, nih or editorial or guideline or historical article or interactive tutorial or interview or introductory journal article or lecture or news or newspaper article or overall or patient education handout or practice guideline or "review" or "scientific integrity review" or systematic review)
13	limit 12 to meta analysis
14	12 not 13
15	11 not 14
16	(tocoly* or Albuterol or Fenoterol or Hexoprenaline or Indomethacin or Isoxsuprine or Magnesium Sulfate or Nifedipine or Nylidrin or Ritodrine or Terbutaline).mp.
17	((multiple adj3 (pregnanc* or birth* or offspring*)) or twin* or triplet* or quintuplet* or quadruplet*).mp.
18	16 and 17
19	MEDLINE.st.
20	18 not 19
21	(biograph* or case report* or comment or congress* or conference* or editor* or tutorial* or interview* or lecture* or news* or handout* or guideline* or (review* not (meta analys* or metaanalys*))).mp.
22	20 not 21
23	or/15,22
24	(trial* or comparative or meta analysis or metaanalysis or multicenter or observational or randomized or randomised or rct or cct or cohort or cross sectional or longitudinal or evaluation or prospective or retrospective or case control).mp.
25	23 and 24
26	23 not 25
27	remove duplicates from 26

Embase (embase.com)

No.	Query
#1	'uterus spasmolytic agent'/exp/mj/dd_do,dd_cm,dd_dt,dd_ad,dd_to,dd_ct,dd_it
#2	'uterus spasmolytic agent'/exp/dd_ae
#3	'tocolysis'/mj
#4	'tocolysis'/dd_ae
#5	#1 OR #2 OR #3 OR #4

#6	'multiple pregnancy'/exp
#7	#5 AND #6
#8	#7 AND 'human'/de
#9	#8 AND [embase]/lim NOT [medline]/lim
#10	'editorial'/de OR 'erratum'/exp OR 'note'/de OR 'review'/de
#11	'meta analysis'/de
#12	#10 NOT #11
#13	#9 NOT #12
#14	'case report'/exp
#15	#13 NOT #14

Cochrane Central Register of Controlled Trials (CENTRAL) (Wiley)

ID	Search
#1	MeSH descriptor: [Tocolytic Agents] explode all trees
#2	MeSH descriptor: [Tocolysis] explode all trees
#3	Tocoly* or Magnesium Sulfate or Ritodrine or Terbutaline or spasmoly*
#4	#1 or #2 or #3
#5	MeSH descriptor: [Pregnancy, Multiple] explode all trees
#6	MeSH descriptor: [Multiple Birth Offspring] explode all trees
#7	"multiple pregnancy":kw
#8	(multiple near (pregnancy or birth)) or twin*
#9	#5 or #6 or #7 or #8
#10	#4 and #9
#11	handsrch
#12	#10 and #11

CINAHL (EBSCO)

ID	Search Terms
S1	(MM "Tocolytic Agents+/AD/DE/TU")
S2	(MH "Tocolytic Agents+/PO/AE")
S3	S1 or S2
S4	(MH "Pregnancy, Multiple+")
S5	(MH "Multiple Birth Offspring+")
S6	S4 or S5
S7	S3 and S6
S8	S7Limiters - Exclude MEDLINE records

WHO Global Index Medicus (Global Index Medicus)

ID	Query/Search terms
1	(tocoly* OR indomethacin* OR "Magnesium Sulfate" OR Ritodrine OR Terbutaline OR spasmoly*) AND (multiple OR twin OR triplet)
